# NsrR, GadE, and GadX Interplay in Repressing Expression of the *Escherichia coli* O157:H7 LEE Pathogenicity Island in Response to Nitric Oxide

**DOI:** 10.1371/journal.ppat.1003874

**Published:** 2014-01-09

**Authors:** Priscilla Branchu, Stéphanie Matrat, Marjolaine Vareille, Annie Garrivier, Alexandra Durand, Sébastien Crépin, Josée Harel, Grégory Jubelin, Alain P. Gobert

**Affiliations:** 1 INRA, UR454 Microbiologie, Centre de Clermont-Ferrand-Theix, Saint-Genès-Champanelle, France; 2 Groupe de Recherche sur les Maladies Infectieuses du Porc and Centre de Recherche en Infectiologie Porcine, Université de Montréal, Saint-Hyacinthe, Québec, Canada; University of Utah, United States of America

## Abstract

Expression of genes of the locus of enterocyte effacement (LEE) is essential for adherence of enterohemorrhagic *Escherichia coli* (EHEC) to intestinal epithelial cells. Gut factors that may modulate LEE gene expression may therefore influence the outcome of the infection. Because nitric oxide (NO) is a critical effector of the intestinal immune response that may induce transcriptional regulation in enterobacteria, we investigated its influence on LEE expression in EHEC O157:H7. We demonstrate that NO inhibits the expression of genes belonging to LEE1, LEE4, and LEE5 operons, and that the NO sensor nitrite-sensitive repressor (NsrR) is a positive regulator of these operons by interacting directly with the RNA polymerase complex. In the presence of NO, NsrR detaches from the LEE1/4/5 promoter regions and does not activate transcription. In parallel, two regulators of the acid resistance pathway, GadE and GadX, are induced by NO through an indirect NsrR-dependent mechanism. In this context, we show that the NO-dependent LEE1 down-regulation is due to absence of NsrR-mediated activation and to the repressor effect of GadX. Moreover, the inhibition of expression of LEE4 and LEE5 by NO is due to loss of NsrR-mediated activation, to LEE1 down-regulation and to GadE up-regulation. Lastly, we establish that chemical or cellular sources of NO inhibit the adherence of EHEC to human intestinal epithelial cells. These results highlight the critical effect of NsrR in the regulation of the LEE pathogenicity island and the potential role of NO in the limitation of colonization by EHEC.

## Author Summary

Enterohemorrhagic *Escherichia coli* (EHEC) O157:H7 are food-borne pathogens for humans causing bloody diarrhea and, especially in children under five years old, kidney damages leading to death in 5% of cases. Antibiotics are contra-indicated because they are suspected to increase the severity of the disease. Therefore, it is crucial to develop alternative preventive or therapeutic strategies to fight EHEC infection. To reach this goal, a deeper knowledge of host-pathogen interaction is required. A critical step in EHEC infection is the adhesion of bacterial cells to intestinal epithelial cells. In response to the bacterial infection, the host triggers an immune response directed against the pathogen. The current study shows that a main effector of this immune response, nitric oxide (NO), dramatically reduces the capacity of EHEC to adhere to intestinal epithelial cells. We have investigated the molecular mechanisms involved and identified a NO-sensor regulator that controls the expression of the genes required for EHEC adhesion. This finding underlines that NO could be a potential protective factor limiting the development of EHEC-induced diseases and provides a new avenue of investigation for the development of therapeutic strategies against infections with O157:H7 bacteria.

## Introduction

Enterohemorrhagic *Escherichia coli* (EHEC), especially those belonging to the O157:H7 serotype, are foodborne pathogens and healthy rearing animals are the main reservoir. Human infection occurs through the ingestion of contaminated food. This primary infection yields to the development of intestinal disorders, including aqueous or bloody diarrhea. Moreover, EHEC express a cardinal and well-defined virulence factor, the Shiga-toxin (Stx) encoded by genes located in lysogenic lambdoid bacteriophages. Stx is produced in the gut lumen and crosses the epithelial barrier to reach the blood and the target organs including the kidneys. In this context, infected patients may develop life-threatening complications such as the hemolytic and uremic syndrome (HUS), the main cause of renal failure in children in developed countries [1].

EHEC genes carried by the locus of enterocyte effacement (LEE), a chromosomal pathogenicity island organized in 5 operons, encode bacterial factors implicated in the intimate adherence of these bacteria to intestinal epithelial cells [2]. These genes encode a type 3 secretion system (T3SS; LEE1, LEE2, LEE3), a translocon and a syringe (LEE4) that allows bacteria to inject effectors in epithelial cells, such as the LEE5-encoded intimin receptor Tir; moreover, other proteins not carried by the LEE can be translocated by the T3SS into enterocytes [3], [4]. The injected effectors and/or protein of the translocon itself interact with the host signal transduction, leading to actin polymerization and to microvilli effacement [2], to regulation of the innate immune response [5], [6], and to increased electrolyte transport [7]. Regulation of gene expression within the LEE is known to be complex and governed by a large number of influences, including environmental cues or quorum sensing, and involves several specific or global regulators [8], [9]. The first gene of the LEE1 operon, *ler*, encodes a transcriptional regulator that positively regulates the expression of all the other operons [9]–[11]. However a variety of extra-transcriptional mechanisms have also been involved in the regulation of LEE expression, though little detailed mechanistic information is available [12]. GadE (YhiE) and GadX (YhiX) are two main regulators of the acid fitness island involved in acid-resistance (AR) in *E. coli* K12 [13]–[15]. At acidic pH values, GadE and GadX positively regulate the *gadA* and *gadBC* genes, encoding the components of the glutamate-dependent AR. In *E. coli* O157:H7, GadE has acquired additional functions and inversely coordinates expression of AR and LEE genes [16]: It has been proposed that, during passage through the human stomach, GadE protects EHEC by inducing the glutamate-dependent AR system and inhibits the unnecessary expression of the LEE genes, while environmental cues in the intestine lead to downregulation of *gadE* and upregulation of the LEE genes [16]. GadE has been shown to directly bind the *ler* (LEE1) and *sepZ* (LEE2) promoters in vitro [8], but in vivo binding of GadE and the role of GadX have never been investigated.

We have previously shown that nitric oxide (NO) decreases Stx2 synthesis by EHEC O157:H7 at the transcriptional level [17]. This occurs through the inhibition of the SOS response by the NO sensor nitrite-sensitive repressor (NsrR) [17], the key regulator of the nitrosative stress in enterobacteria [18]. In this context, our aim was to investigate whether NO also modulates LEE gene transcription and therefore EHEC adhesion to epithelial cells. Here we show that NsrR is a direct positive regulator of the transcription of LEE1, LEE4 and LEE5 genes and an indirect repressor of *gadE* and *gadX* genes. In the presence of NO, LEE1/4/5 activation is abrogated, GadE is induced and yields to *gadX* expression. Finally, we identify GadE and GadX as repressors of LEE4/5 and of LEE1, respectively. Using a human intestinal epithelial cells/EHEC co-culture model we demonstrate that bacterial adhesion is inhibited in NO producing cells.

## Results

### The adhesion of EHEC to intestinal epithelial cells is reduced by NO

**Figure 1:**
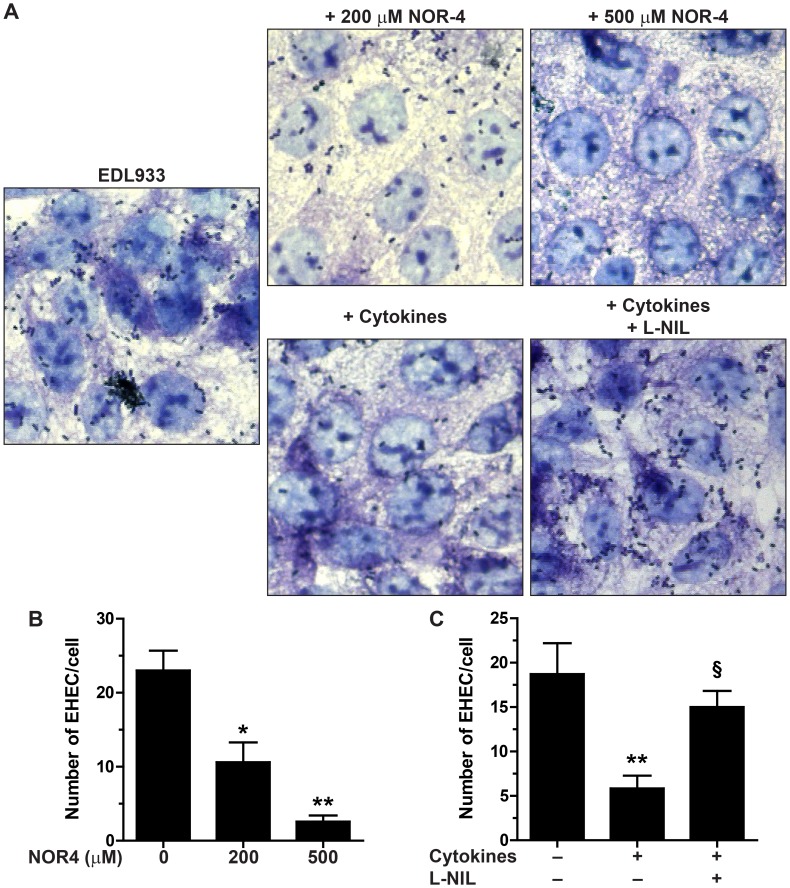
Adhesion of EDL933 to intestinal epithelial cells. Hct-8 cells, pre-treated or not with a cytokine cocktail for 24 h, were co-cultured for 6 h with the EHEC strain EDL933 ± NOR-4 or L-NIL. A: Cells and bacteria were visualized after Giemsa staining; magnification, ×63. B and C: The number of bacteria adherent per Hct-8 cell was counted on 15 microscopic fields. For B, * *P*<0.05, ** *P*<0.01 compared to the co-cultures without NOR-4; n = 6. For C, ** *P*<0.01 vs. cells not stimulated with cytokines; § *P*<0.05 vs. cells treated with cytokines; n = 6.

We first examined adhesion of the *E. coli* O157:H7 strain EDL933 to cultured Hct-8 intestinal epithelial cells in the presence of the NO donor NOR-4. Exposure to NOR-4 at 200 µM or 500 µM did not cause any significant difference in the growth rate of EDL933, as described [17]. However, EHEC adhesion to Hct-8 cells was dramatically inhibited when NOR-4 was added to the co-cultures (Figs. 1A and 1B). The number of EHEC fixed to the cells was significantly decreased by 41±5% and 89±2% in the presence of 200 µM and 500 µM NOR-4, respectively (Fig. 1B).

To further confirm this result, we analyzed the effect of endogenous NO released by enterocytes. Hct-8 cells were first treated for 24 h with a cytokine cocktail known to stimulate the inducible NO synthase (iNOS) expression [19], washed, and then infected with the strain EDL933 in the presence or absence of the iNOS inhibitor N^6^-(1-iminoethyl)-l-lysine (l-NIL). There was less EHEC fixed to NO-producing epithelial cells than to control cells (Figs. 1A and 1C). The inhibition of EHEC adherence to Hct-8 cells treated with cytokines was abolished by the use of l-NIL (Figs. 1A and 1C).

### NO inhibits LEE1/4/5 gene expression and stimulates the Gad system

**Figure 2:**
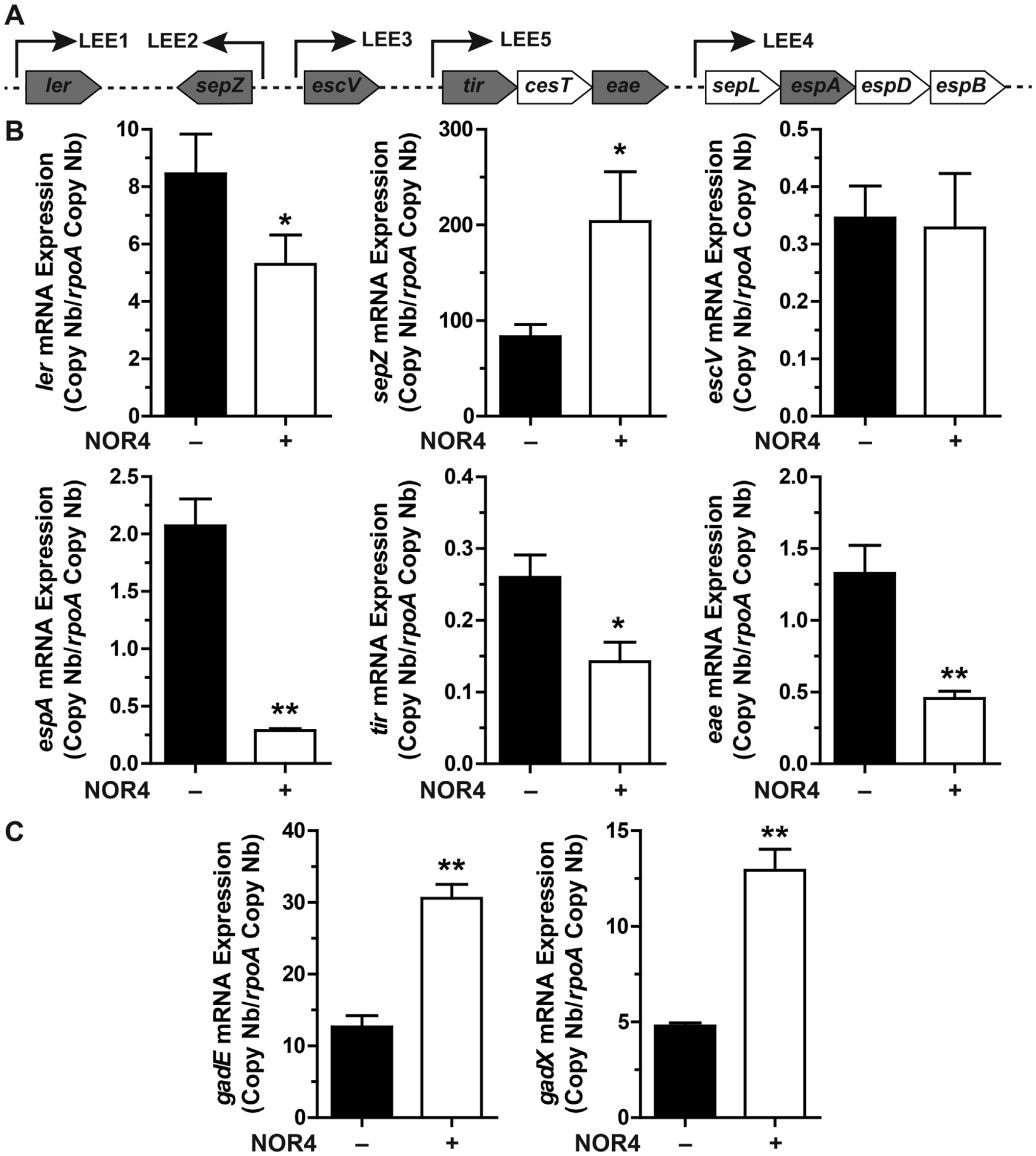
Influence of NO on LEE, *gadX*, and *gadE* gene expression. A: schematic representation of the LEE showing the structural organization of the main operons. Arrows indicate the orientation of the transcription. The genes analyzed in this study are in grey boxes. B and C: EDL933 was grown for 6 h with or without NOR-4. The expression of the LEE genes (B) and of *gadE* and *gadX* (C) was analyzed by RT-qPCR. * *P*<0.05, ** *P*<0.01 compared to the strain grown in the absence of NOR-4; n = 3–6.

The expression of genes that represent the five operons of the LEE (Fig. 2A) was analyzed after treatment with NOR-4 for 6 h. NO was consistently generated in the bacteria culture medium and reached a plateau after 6 h (Fig. S1). The expression of *ler* (LEE1), *espA* (LEE4), *tir* and *eae* (LEE5) was down-regulated by NO, while the transcription of *sepZ* (LEE2) was induced by 2.4-fold (Fig. 2B). The expression of the gene *escV* (LEE3) was not modulated by NOR-4 (Fig. 2B). Because GadE and GadX modulates LEE expression in EHEC and EPEC, respectively, [16], [20], we investigated the effect of NOR-4 on *gadE* and *gadX* transcription. As shown in Figure 2C, the expression of *gadE* and *gadX* was significantly induced by 2.4- and 2.7-fold in bacteria exposed to NOR-4, respectively. Thereby, these data prompted us to wonder whether NO-dependent down-regulation of LEE1, LEE4 and LEE5 requires GadE and/or GadX.

### GadE and GadX modulate the expression of LEE genes

**Figure 3:**
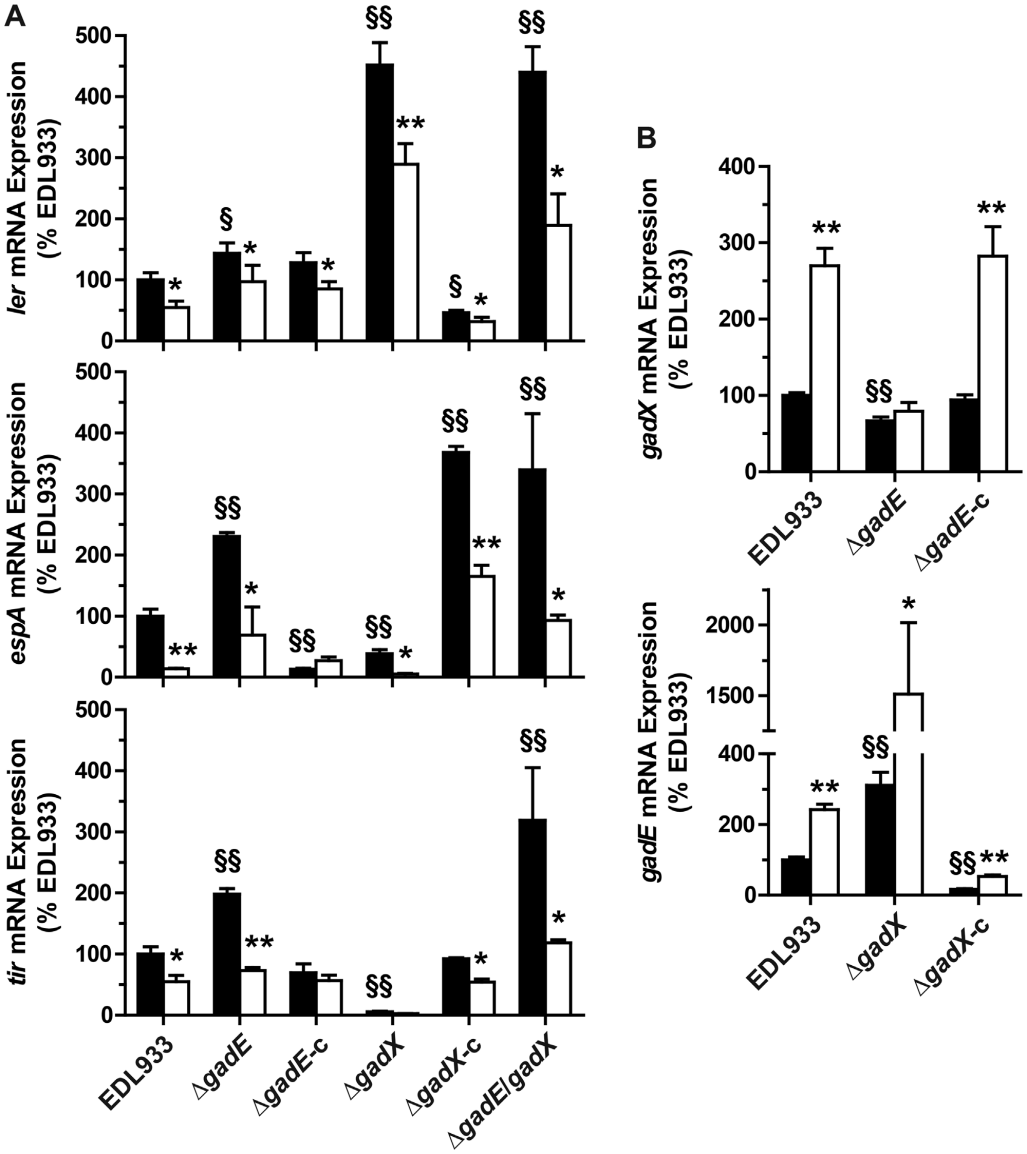
Regulation of LEE1/4/5 by GadE and GadX. The mRNA levels of *ler* (LEE1), *espA* (LEE4), and *tir* (LEE5) (A) and of *gadE* and *gadX* (B) were assessed in the strain EDL933, in the Δ*gadE*, Δ*gadX* and Δ*gadE*/*gadX* isogenic mutants, and in the complemented strains Δ*gadE*-c and Δ*gadX*-c. Bacteria were grown in the absence (black bars) or presence (white bars) of NOR-4 for 6 h. * *P*<0.05, ** *P*<0.01 compared to the same strain without NOR-4; § *P*<0.05, §§ *P*<0.01 vs. EDL933; n = 3–7.

Since the role of GadX and GadE on LEE expression is not well defined and is strongly dependent on the growth conditions [16], [20], [21], we first analyzed the expression of *ler*, *espA*, and *tir* in EDL933 Δ*gadE* and Δ*gadX* mutants. When compared to the EDL933 strain, the mRNA levels of *ler, espA* and *tir* were increased by ∼1.4-, 2.3-, and 2-fold in the Δ*gadE* strain, respectively (Fig. 3A); these effects were reversed when the *gadE* mutant was trans-complemented with the *gadE* gene in a low copy number plasmid vector (Fig. 3A). The *gadX* mutation was associated with a spontaneous increase of *ler* transcription and with a significant reduction of *espA* and *tir* gene expression (Fig. 3A). The transcription of *ler* was repressed while the expression of *espA* was activated and that of *tir* was restored to the same level as the WT in the trans-complemented strain (EDL933 Δ*gadX*-c; Fig. 3A). These data suggest that GadE represses the expression of LEE4 and LEE5 genes independently of Ler, and that GadX represses LEE1 but activates LEE4 and LEE5 gene expression. Interestingly, the NOR-4-dependent down-regulation of *ler*, *espA*, and *tir* was still observed in the Δ*gadE*, Δ*gadX* and Δ*gadE*/*gadX* mutants (Fig. 3A), suggesting that another factor is implicated in the inhibition of LEE1/4/5 by NO.

We next wonder whether GadE and GadX repressed the LEE independently from each other or whether GadX is epistatic to GadE as in *E. coli* K12 [22]. The expression of *ler* was similar in a Δ*gadE*/*gadX* double mutant and in the EDL933 Δ*gadX* strain (Fig. 3A), indicating that GadX is epistatic to GadE in controlling LEE1. Conversely, *espA* and *tir* mRNA levels were increased in EDL933 Δ*gadE*/*gadX* when compared to the WT strain, as in the Δ*gadE* strain, demonstrating that GadE is epistatic to GadX for the regulation of LEE4 and LEE5. Therefore we investigated whether GadE controls *gadX* expression. Figure 3B shows a 33% decrease in *gadX* mRNA levels in the *gadE* mutant, indicating that GadE activates *gadX* expression. In addition, we observed 3.1-fold more *gadE* mRNA copies in the Δ*gadX* strain than in the WT strain (Fig. 3B) and *gadE* mRNA levels were dramatically reduced in the complemented strain (Fig. 3E), suggesting that GadX is a repressor of *gadE* expression. Therefore, the moderate increase in *ler* expression observed in the Δ*gadE* strain (Fig. 3A) is likely due to the lower level of GadX in this strain and not to a direct effect of GadE on *ler* transcription. Lastly, the activation of *gadX* transcription by NOR-4 was suppressed in the *gadE* mutant, but not in the EDL933 Δ*gadE*-c strain (Fig. 3B), while the NO-dependent induction of *gadE* mRNA expression was still observed in the Δ*gadX* strain (Fig. 3B). These data indicate that NO activates *gadX* expression through GadE.

### The repression of LEE1/4/5 genes is mediated by NsrR

**Figure 4:**
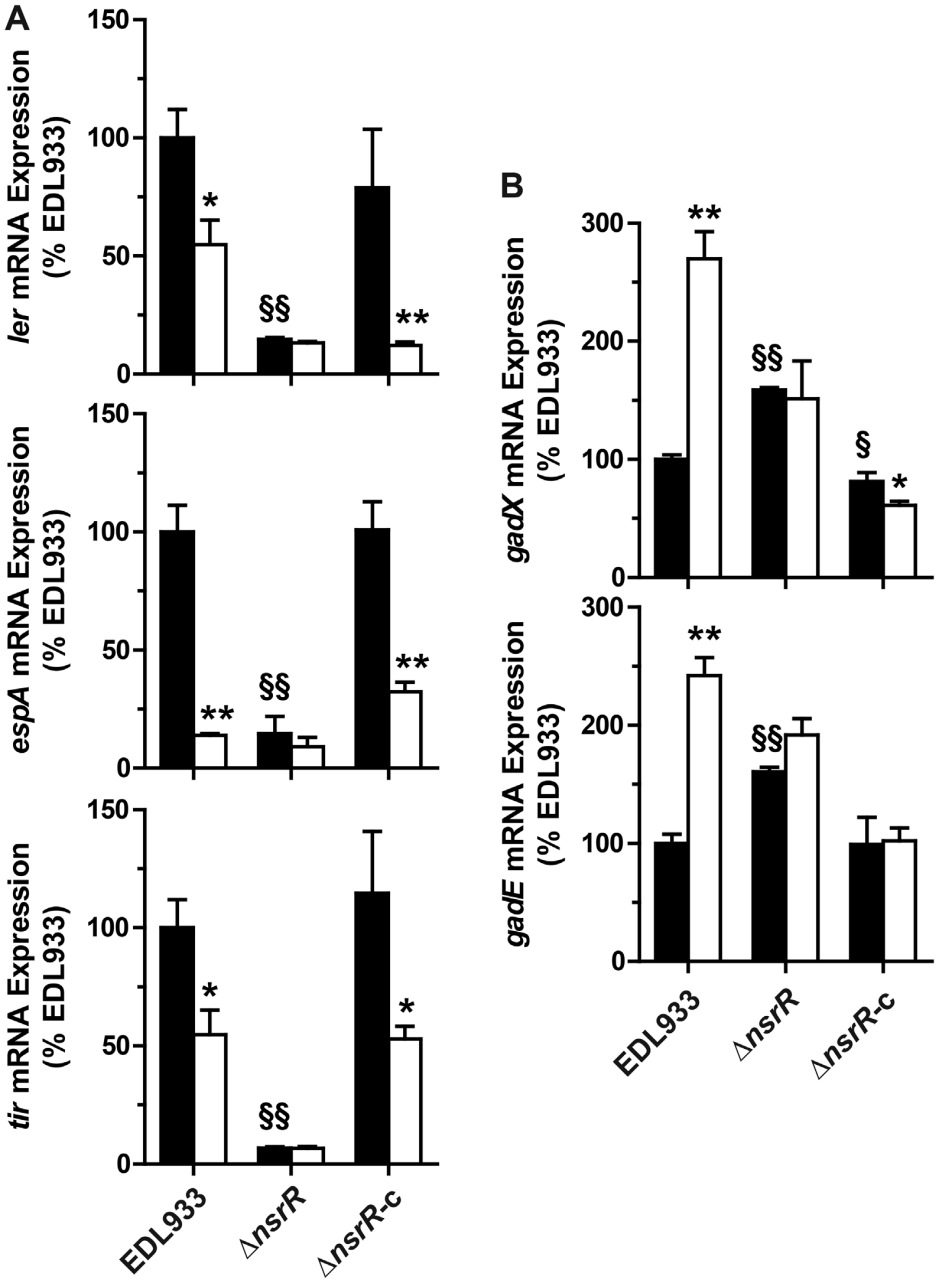
Effect of NsrR on LEE, *gadX*, and *gadE* gene expression. The strains EDL933, Δ*nsrR*, or Δ*nsrR*-c were grown with (white bars) or without (black bars) NOR-4. The expression of the genes *ler*, *espA*, *tir* (A) and *gadX* and *gadE* (B) was analyzed. * *P*<0.05, ** *P*<0.01 compared to the same strain without NOR-4; § *P*<0.05, §§ *P*<0.01 vs. EDL933; n = 3.

NsrR is a transcriptional regulator that regulates gene expression in response to NO [18]. Therefore we investigated whether NsrR regulates *gadE*, *gadX*, and the LEE genes. In the absence of NO, the mRNA levels of *ler*, *espA*, and *tir* were 6.8, 7.1, and 14.3-fold lower in the Δ*nsrR* mutant than in the WT strain, respectively (Fig. 4A). The expression of these genes was similar in the strains EDL933 and EDL933 Δ*nsrR*-c (Fig. 4A). Moreover, the NO-dependent regulation of these LEE genes was abrogated in EDL933 Δ*nsrR* and was restored in the complemented strain (Fig. 4A). Inversely, the transcription of *gadE* and *gadX* was significantly increased in the Δ*nsrR* mutant, but not in the complemented strain. The expression of these two genes was not affected by NOR-4 in the *nsrR*-deficient strain (Fig. 4B).

These data suggest that NsrR is a transcriptional activator of LEE1, LEE4, and LEE5 and a repressor of *gadE*, which in turn modulates *gadX* expression. NsrR loses its ability to regulate the expression of LEE and *gad* genes in the presence of NO.

### The NsrR binding on the promoter regions of LEE 1/4/5 is inhibited by NO

The investigation of GadE, GadX and/or NsrR direct binding to the *gadE*, *gadX*, and LEE promoter regions was performed by chromatin immunoprecipitation (ChIP) experiments using the EDL933 Δ*gadE*, Δ*gadX*, and Δ*nsrR* mutants expressing the 6-His-GadE, the 6-His-GadX, and the 6-His-NsrR fusion proteins, respectively.

**Figure 5:**
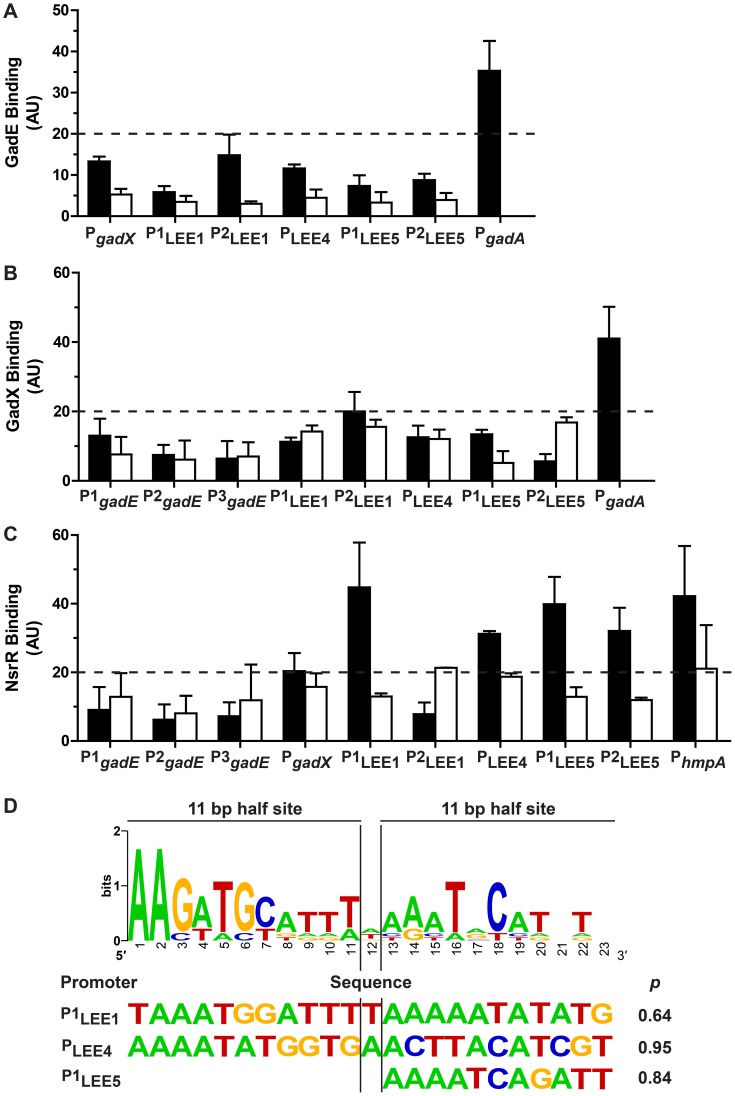
Binding of GadE, GadX and NsrR on various promoter regions. The strains EDL933 Δ*gadE*-pBADMycHisA::*gadE*, Δ*gadX*-pBADHisA::*gadX*, and Δ*nsrR*-pBADMycHisA::*nsrR* were grown in the absence (black bars) or in the presence (white bars) of NOR-4. ChIP assays followed by qPCR were performed to determine the relative enrichment in DNA molecules bound to GadE (A), GadX (B), NsrR (C). Values higher than 20 (twice the values obtained for the strain EDL933 containing the empty pBADmycHisA vector) indicate protein binding to the promoters of interest. D: Bio-informatics analyses of NsrR-binding sites. Sequence logo determined from seven putative NsrR-binding sites in EDL933 (upper panel), and sequences with the best matches for the entire or one of the half sites are shown with their statistical scores (lower panel).

We first analyzed the *gadX* promoter described by Hommais *et al.* [15], the three promoters described for *gadE* in *E. coli* K12 [22] (Fig. S2), and the *gadA* promoter as a positive control for GadE and GadX binding [23]. Surprisingly, we found that GadE and GadX did not bind to the *gadX* and *gadE* promoters, respectively (Figs. 5A and 5B), indicating that activation of *gadX* by GadE and repression of *gadE* by GadX occur through indirect regulations. As expected, the binding of GadX and GadE to the *gadA* promoter region was observed (Figs. 5A and 5B).

Two *ler* promoters have been described in EHEC, the distal P1 promoter and a putative proximal P2 promoter (Fig. S2). The P1 promoter is common to EHEC and EPEC, while the P2 promoter is present only in EHEC [24]–[26]. Neither GadE (Fig. 5A) nor GadX (Fig. 5B) bound to either of these promoters (Figs. 5A and 5B). These data indicate that GadE and GadX do not repress *ler* expression directly. The LEE4 promoter has been identified in EHEC upstream of *sepL* [27], *espA* being the second gene of the operon. In EPEC, it has been shown that Ler-mediated activation of the LEE5 operon requires sequences between positions -198 and -75 relative to the transcriptional start site [28]. Two primer pairs overlapping this region have been designed for ChIP experiments, amplifying a LEE5 distal (P1_LEE5_) and a LEE5 proximal (P2_LEE5_) region (Fig. S2). ChIP experiments showed that neither GadE nor GadX bound to the LEE4 and LEE5 promoters (Figs. 5A and 5B). Lastly, the binding of GadE and GadX to the LEE1/4/5 promoter regions was not modulated by NOR-4. These data indicate that control of LEE4 and LEE5 expression by GadE and GadX is due to indirect effects.

In contrast, NsrR bound to the distal LEE1 promoter (P1_LEE1_), to the LEE4 and LEE5 promoters, and to the promoter of *hmpA*, a well-know NsrR target gene (Fig. 5C). Furthermore, NsrR binding to these promoter regions was inhibited when the bacteria were grown in the presence of NOR-4 (Fig. 5C). We did not observed NsrR binding to the *gadE* and *gadX* promoters (Fig. 5C).

We thus performed bio-informatics analysis to identify putative NsrR-binding sites in the LEE1, LEE4 LEE5, *gadE*, and *gadX* promoters in the strain EDL933. We used the homologous sequences of seven NsrR-binding sites described in *E. coli* K12 [29] to generate the sequence logo of the NsrR box in the strain EDL933 (Fig. 5D). We then performed bioinformatics analysis on the LEE1, LEE4 and LEE5 promoter sequences by the Gibbs Sampler Motif Software, using the matrix of the seven putative NsrR-binding sites of EDL933. In agreement with the ChIP data, bioinformatics analysis identified sequences presenting high identity with the NsrR consensus binding site in the LEE1 (P1), LEE4 and LEE5 promoter regions (Fig. 5D and Fig. S2), but not in the promoters of *gadE* and *gadX*. The analysis indicated a 23 bp putative NsrR-binding site in the promoters of LEE1 (86.9% identity) and LEE4 (78.2% identity), but only a second half-site NsrR-binding site in the LEE5 promoter (90.9% identity for the half site; Fig. 5D). *In silico* analyses performed using the BLAST program indicated that these putative binding sites are conserved in a number of EHEC and EPEC strains, but not in *Citrobacter rodentium* (Fig. S3), an attaching/effacing pathogen that infects rodents.

### NsrR interacts with the RNA polymerase complex

**Figure 6:**
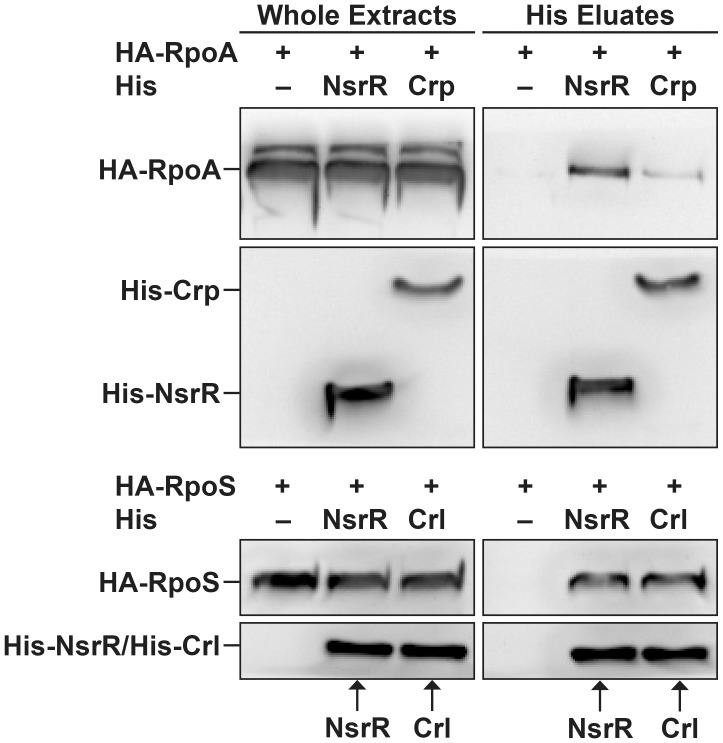
NsrR interacts with RNA polymerase complex. His-NsrR, His-Crp or His-Crl proteins were co-expressed in bacteria with HA-RpoA or HA-RpoS as indicated. His tagged proteins were then purified from bacterial lysates using nickel affinity. Whole extracts (1 µg) and His eluted fractions (5 µl) were probed with anti-His or anti-HA Abs.

Since NsrR has been exclusively described as a transcriptional repressor, we investigated the molecular mechanism underlying the direct activation of LEE gene expression by NsrR. For many transcriptional activators, increase of the transcription level results from the recruitment of RNA polymerase through direct interaction between the regulatory protein and one or several subunits of the polymerase [30]. We therefore examined if NsrR can interact with α and σ RNA polymerase subunits. To this end, His-tagged NsrR and hemagglutinin (HA)-tagged polymerase subunits α (RpoA) or σ^38^ (RpoS) were co-expressed in bacteria. His-NsrR was purified under native conditions using a nickel affinity resin and the different fractions were analyzed by western-blot. As positive controls, RpoA and RpoS were also co-expressed with His-Crp or His-Crl, respectively, two well-known interacting partners [31], [32]. All tagged proteins were properly expressed as revealed by their immunodetection in the whole extract samples (Fig. 6). As expected, HA-RpoA and HA-RpoS co-eluted with His-Crp or His-Crl, respectively. No HA-tagged protein was detected in the His eluates of the negative controls, i.e., bacteria expressing only HA-tagged proteins (Fig. 6). Importantly, HA-RpoA and HA-RpoS were also specifically recovered in the eluted fractions from the His-NsrR purifications. This finding demonstrates that NsrR can interact with the RNA polymerase complex and suggests that NsrR activates LEE gene expression through the recruitment of RNA polymerase.

### Adhesion of the regulatory mutants to HeLa cells

**Figure 7:**
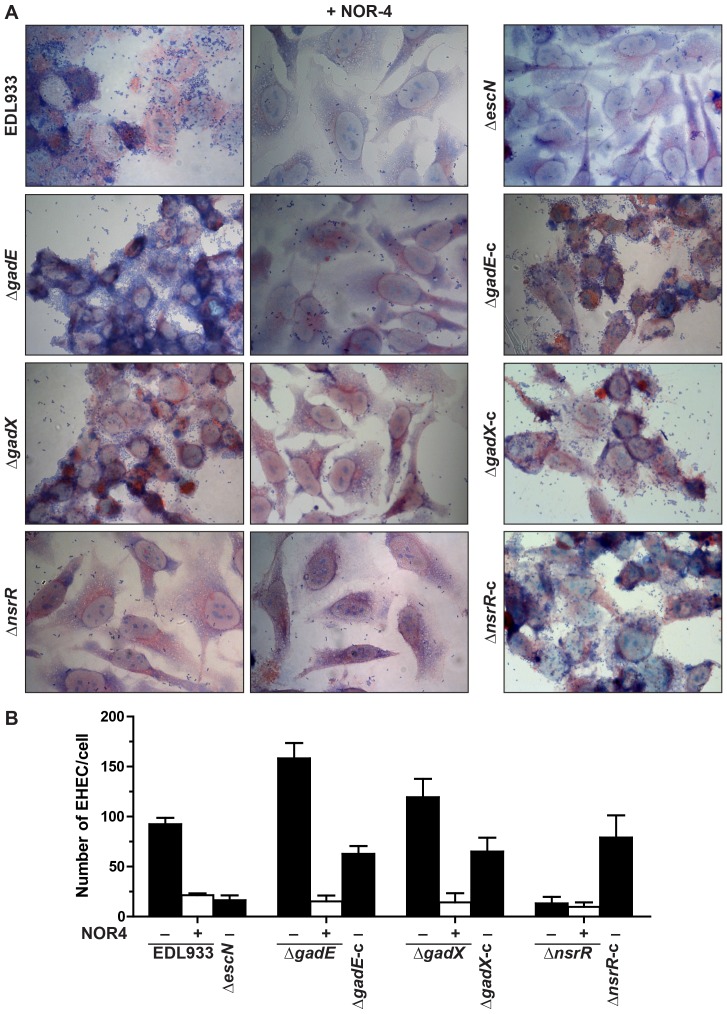
Regulation of adhesion of EDL933 to human epithelial cells. HeLa cells were infected with EDL933, Δ*gadE*, Δ*gadX*, Δ*nsrR*, or with the corresponding trans-complemented strains, in the presence or absence of NOR-4. After 6 h, cells were washed and colored with May-Grünwald Giemsa (A). The number of adherent bacteria per Hela cell was determined from 50 cells (B).

In order to confirm the role of NO, NsrR, GadE, and GadX in regulating LEE expression, we investigated the attachment of the regulatory mutants to HeLa cells after 6 h of infection in the presence or absence of NOR-4. As expected, EDL933 adhered to HeLa cells and when NOR-4 was added to the co-culture the level of adhesion was dramatically reduced to that of the Δ*escN* mutant that lacks a functional T3SS (Figs. 7A and 7B). The adhesion of the Δ*gadE* and Δ*gadX* strains was higher than that of the parent strain, correlating with the repressive effect of AR regulatory proteins on LEE gene expression (Figs. 7A and 7B). Conversely, the *nsrR* mutant was less adherent than the WT strain (Figs. 7A and 7B). The complementation of these three mutants restored the adhesion phenotype of the parental strain (Figs. 7A and 7B). Under NO exposure, adherence properties were affected for the Δ*gadE* and Δ*gadX* mutants but not for the Δ*nsrR* mutant (Figs. 7A and 7B), demonstrating that NsrR is the key regulator controlling the T3SS-dependent adhesion of EHEC in response to NO.

## Discussion

**Figure 8:**
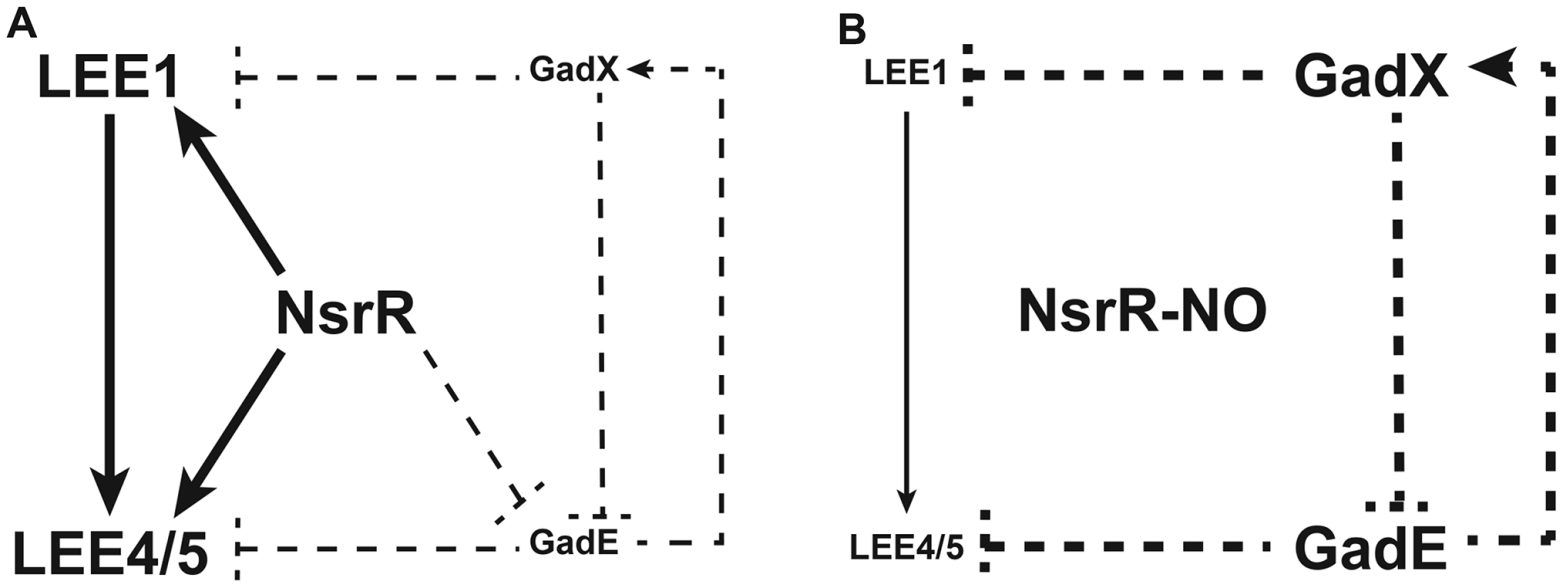
A model for the NO-dependent regulation of LEE1/4/5. Solid lines indicate physical interaction between the regulator and the promoter as demonstrated by ChIP; dotted lines indicate that no physical interaction has been demonstrated between the regulator and the promoter. A: In the absence of NO, NsrR directly activates LEE1, LEE4, and LEE5 expression and indirectly represses *gadE* and therefore *gadX* expression. GadE activates *gadX* expression and acts as an indirect repressor of LEE4 and LEE5. GadX is an indirect repressor of *gadE* and LEE1 expression. B: Under NO exposure, NO binds to NsrR, which is consequently released from its target DNA. Thus, the activation of LEE1/4/5 genes by NsrR is suppressed. In parallel, *gadE* expression is restored and induces *gadX* up-regulation. In this context, GadE strongly represses LEE4 and LEE5 genes while GadX inhibits LEE1 expression.

In the present report, we show that NO, a critical mediator of the host innate immune response, is a potent inhibitor of LEE gene expression in EHEC O157:H7 and consequently inhibits the adhesion of these pathogens to intestinal epithelial cells. We identified NsrR as an unrecognized regulator that controls the expression of LEE genes in response to NO, and we propose a regulatory model presenting the role of NsrR, GadE and GadX in LEE expression (Fig. 8). In the absence of NO (Fig. 8A), NsrR directly activates LEE1, LEE4, and LEE5 gene expression, and indirectly represses *gadE* and therefore *gadX* expression. We also show that GadE indirectly activates *gadX* expression and represses LEE4 and LEE5 expression independently of Ler, while GadX inhibits *gadE* and LEE1 expression. When NsrR binds NO (Fig. 8B), it is released from its target DNA, leading to loss of induction of LEE1/4/5 genes and to the up-regulation of *gadE* and, consequently, *gadX*. In this context, the NO-dependent LEE1 down-regulation is due to absence of NsrR-mediated activation and to the inhibitory effect of GadX. In parallel, the inhibition of LEE4 and LEE5 gene expression is due to absence of NsrR- and Ler-dependent activation and to increase of GadE level. This model assumes that repression of *gadX* expression by NsrR is mediated by GadE, which is consistent with the observation that the NO-dependent activation of *gadX* is abrogated in the Δ*gadE* and Δ*nsrR* mutants.

NsrR is a key negative regulator of the nitrosative stress in enterobacteria [18], [33]. NsrR has always been described as a transcriptional repressor. In addition, its DNA-binding activity is suppressed in the presence of NO, yielding to the expression of various genes mainly involved in NO detoxification [18], [33]. In non-pathogenic *E. coli*, NsrR also regulates expression of genes involved in metabolism, motility, protein degradation, surface attachment, stress response and transmembrane transport [29], [34]. Our data indicate that NsrR is also a repressor of the genes *gadE* and *gadX*. Nonetheless, the NsrR-dependent repression of *gadX* is probably mediated by GadE since the NO-dependent up-regulation of *gadX* is abrogated in the Δ*gadE* mutant. We did not find a sequence matching the NsrR consensus binding site in the *gadE* promoter, and ChIP experiments failed to demonstrate physical interaction between NsrR and the *gadE* promoter. Therefore, the effect of NsrR on *gadE* transcription is probably indirect and mediated by an unknown regulatory cascade controlled by NsrR.

Here we provide compelling evidence that NsrR is a direct positive regulator of LEE1, LEE4, and LEE5 operons in EHEC by binding to their own promoters. Moreover, our data also suggest that NsrR acts as a transcriptional activator by recruiting RNA polymerase to promoter regions since NsrR is able to pull-down the α and σ^38^ subunits of the RNA polymerase. Supporting the concept that it may also be a transcriptional activator, it has been reported that NsrR activates virulence gene expression in *Salmonella* Typhimurium, in particular expression of genes important for eukaryotic cell adherence, invasion and intestinal translocation, and that an *nsrR* mutant is impaired in invasion of HeLa cells [35]. However, *in silico* analysis failed to identify an NsrR consensus binding site in the promoter regions of these genes, indicating that the positive regulatory effect of NsrR is probably indirect in this pathogen [35]. Moreover, using an *E. coli* K12 strain harboring a multicopy plasmid that titrates out NsrR, Filenko *et al.* have identified by a microarray analysis 22 transcripts that could be directly or indirectly activated by NsrR [34].

The NsrR binding site is a 23 bp palindrome sequence composed of two 11 bp half sites separated by a single nucleotide, and NsrR binds to DNA as a dimer [36]. However, a number of NsrR target promoters contain only a single half site [29]. Potential NsrR consensus sequence were identified in the LEE1, LEE4 and LEE5 promoters, with a 23 pb putative NsrR-binding site in the LEE1 and LEE4 promoters, and a putative second half-site in the LEE5 promoter. It has been suggested that, when the NsrR binding site contains only a single half site, one NsrR monomer makes specific contact to the consensus half site and the other monomer forms nonspecific contact [37]. Alternatively, it has been suggested that NsrR binds as a tetramer to the complete binding motif and as a dimer when only one half site is conserved [29]. It is noteworthy that the putative NsrR binding sites identified in the LEE1, LEE4 and LEE5 promoters are conserved in a number of other EHEC and EPEC strains, but not in *C. rodentium*, suggesting that NO also influences cell adhesion *via* NsrR in other *E. coli* attaching/effacing pathogenic human strains.

Influence of GadE on LEE gene expression remains controversial. While Tatsuno *et al.* described an increased expression of LEE2, LEE4, and LEE5 in a Δ*gadE* mutant, which is not correlated with enhancement of *ler* expression [20], KailasanVanaja *et al.* showed that GadE represses LEE expression by down-regulating *ler* transcription [16]. These discrepancies are proposed to be due to differences in growth medium and/or differences in the sensitivity of the assays used in each study. Interestingly, our data indicate that GadE may repress the expression of LEE4 and LEE5 *via* two regulatory cascades, mediated or not by Ler (Figure 8). On the one hand, we show that GadE inhibits LEE1 through GadX, because a decreased expression of *gadX* and an induction of LEE1 are observed in the *gadE*-deficient strain; this results in loss of Ler-dependent induction of LEE4/5. On the other hand, the deletion of *gadX* is associated with an increased expression of *ler* and *gadE*, and with an inhibition of LEE4/5, suggesting that GadE inhibits these operons independently of Ler. In accordance, the induction of *espA* and *tir* in the *gadE* mutant and in the Δ*gadE*/*gadX* strain demonstrates that GadX regulates LEE4/5 via the repression of *gadE*. However, although it has been shown in vitro that GadE can bind to the *ler* promoter in EHEC O157:H7 [8], we did not observe such an interaction in vivo in our experiments; this difference is probably due to the presence of binding competitors in live bacteria. Regarding GadX, we show herein that it negatively regulates *ler* transcription in EHEC. However, the effect of GadX on LEE1 expression is indirect since no physical interaction between GadX and the LEE1 promoter has been demonstrated. Interestingly, it has been described in EPEC that LEE1 is down-regulated under conditions in which GadX is induced, namely at pH 5.5 or in contact to epithelial cells [21]; this occurs through the inhibition of the transcription of the *per* locus by GadX [21]. Because the *perC* homologue in EHEC, named *pch*, is involved in LEE1 induction [38], it would be interesting to now determine the role of GadX on *pch* expression.

The biological relevance of LEE1, LEE4, and LEE5 inhibition by NO is the decreased adhesion of *E. coli* O157:H7 to epithelial cells. When EHEC are ingested with the contaminated food, they first reach the stomach. It has been proposed that the acidic conditions of this ecological niche favor GadE induction and therefore limit EHEC adhesion to gastric tissues [16]. There is also abundant nonenzymatically formed NO in the gastric juice caused by acidification of nitrate and nitrite. In this context, we now propose that the NO-dependent LEE4/5 inhibition is a supplementary mechanism developed by EHEC to avoid their persistence in the stomach and to favor bacterial colonization in the colon. Moreover, we have shown in the present study that, not only a chemical source of NO, but also the reactive nitrogen species released by iNOS-expressing colonic epithelial cells inhibit the adherence of O157:H7 *E. coli*, and our previous work has identified NO as a potent inhibitor of Stx synthesis [17]. Together, these results suggest that NO might limit the infectious process and HUS development. Nonetheless, it has been described that EHEC inhibit the inducible transcription of iNOS in human enterocytes [19], thus, by limiting NO production, EHEC might favor their own virulence by increasing the intimate adherence to the intestinal epithelium and Stx synthesis. We can therefore speculate that the issue of the crosstalk between EHEC and the host-derived NO might determine the outcome of the infection.

## Materials and Methods

### Bacteria, mutagenesis, and growth conditions

Strains and plasmids used in this study are listed in Table S1. The EHEC O157:H7 strain EDL933 [39] was used throughout the study. The EDL933 Δ*gadE* and Δ*gadX* mutants and the Δ*gadE*/*gadX* double mutant were constructed using the one-step PCR-based method [40], [41]. Mutants were verified by PCR to assess the loss of the gene and by RT-qPCR to confirm lack of expression of the gene of interest, using the primers listed in Table S2. The Δ*nsrR* mutant strain has been previously described [17]. For complementation analysis and ChIP experiments, the *gadE*, *gadX*, and *nsrR* genes were amplified with the high fidelity polymerase Pfx50 (Invitrogen) and cloned under the control of the *araC* promoter into a low-copy plasmid containing a 6-histidine tag (pBADHisA or pBADMycHisA; Invitrogen), or in pBAD33. The cloned genes were checked by nucleotide sequencing, and their expression was analyzed by RT-qPCR. The 6-His-NsrR-, 6-His-GadE-, and 6-His-GadX-encoding genes were expressed at the same level than the WT genes. To verify the mutation of the *gadE* and *gadX* genes, we analyzed the acid resistance of the mutant strains [42]: Acid-resistance of the Δ*gadE* and Δ*gadX* mutants dropped to 0 and 1.41% of the parent strain, respectively; acid resistance was restored in the complemented mutant strains (data not shown).

A single colony of EDL933 or isogenic mutants was grown overnight in DMEM Low glucose containing 10 mM HEPES. These cultures were diluted in fresh medium to an OD_600_ = 0.03 and grown at 37°C. The medium was supplemented with ampicillin (50 µg/ml), kanamycin (50 µg/ml), chloramphenicol (25 µg/ml), L-arabinose (0.1 mM–0.5 mM), or the NO donor NOR-4 (Enzo Life Science) when required.

### Bioinformatics analysis of NsrR-binding sites

The NsrR-binding sequence logo of the strain EDL933 was generated using homologous sequence of the seven NsrR-binding sites described previously by Partridge *et al.* in *E. coli* K-12 strain MG1655 [29] and the software Weblogo (http://weblogo.berkeley.edu/logo.cgi). The probabilities of occurrence matrix from the seven homologous sequences in EHEC O157:H7 strain EDL933 served as a model for the identification of a consensus sequence in the promoter regions of the target genes using the online software Gibbs Motif Sampler (http://ccmbweb.ccv.brown.edu/gibbs/gibbs.html). The sequence alignment of the LEE1, LEE4 and LEE5 putative sites in other EHEC strains, in EPEC strains, and in *C. rodentium* was performed with the MEGA5 software.

### ChIP assay

The pBADMycHisA::*gadE*, pBADHisA::*gadX*, and pBADMycHisA::*nsrR* plasmids, encoding 6His-GadE, 6His-GadX and 6His-NsrR, were electroporated into the respective mutants to avoid native protein interference. Overnight cultures of each strain in LB medium were diluted 1∶100 in 25 ml of fresh DMEM medium buffered with 10 mM HEPES, with or without NOR-4. GadE and GadX expression was induced with 0.5 mM l-arabinose and NsrR with 0.1 mM l-arabinose. After 6 h of growth with shaking, ChIP was performed as described by Lannois *et al.* [43] with slight modifications. First, the protein-DNA complexes were cross-linked by treating bacteria with 1% formaldehyde at room temperature for 30 min. Bacteria were then washed twice with cold PBS and incubated for 30 min at 37°C in 0.7 ml of lysis buffer (10 mM Tris pH 8, 50 mM NaCl, 10 mM EDTA, and 20% sucrose) containing 10 mg/ml lysozyme (Sigma). Then, 0.7 ml of 2X IP buffer (100 mM Tris pH 8, 300 mM NaCl, 2% Igepal CA-630, 0.5% Na deoxycholate) containing 1 mM PMSF was added and samples were incubated 15 min at 37°C, cooled down on ice, sonicated, and incubated on ice for 1 min. Sonication was repeated 11 times to obtain a solution of fragmented chromatin. A 50 µl aliquot of each sample was treated with 100 µl TE containing 36 µg proteinase K for 2 hours at 37°C, incubated 8 hours at 67°C to reverse crosslinking, and the DNA was purified with the kit Qiaquick (Qiagen); this was termed as Input fraction. The rest of the fragmented chromatin was used to generate the IP fraction. After a 2 h-incubation with an anti-Histidine monoclonal antibody (Sigma), protein G sepharose 50% (40 µl) was added to each sample and incubated 1 hour at room temperature. The beads were washed twice with IP buffer, twice with 1 ml of ChIP wash buffer (10 mM Tris HCl pH 8, 250 mM LiCl, 1 mM EDTA, 0.5% Igepal CA-630, and 0.5% Na deoxycholate) and twice with 1 ml of TE buffer. The beads were resuspended in 100 µl of elution buffer (50 mM Tris HCl pH 8, 10 mM EDTA, 1% SDS), incubated 15 min at 65°C, and centrifuged at 9500× g for 1 min. The supernatants containing the immunoprecipitated DNA were collected and incubated with 100 µl TE containing 36 µg proteinase K 2 hours at 37°C and 8 hours at 65°C. DNA was purified with the Qiaquick kit (Qiagen) and amplified by qPCR using the primers listed in Table S2 and depicted in Fig. S2.

The enrichment of DNA targets was calculated as follows for each protein: the promoters of interest as well as a non-specific *rpoA* intragenic region were amplified with specific primers (Table S2). For each DNA target, we calculated the ratio between the copy number in the IP fraction and the Input fraction; each value was then divided with the ratio obtained for the non-specific *rpoA* intragenic region. Then the same ratio was calculated from the parent strain EDL933 containing the empty pBADMycHisA vector. Values higher than 20, corresponding to twice the values obtained for the strain EDL933 containing the empty pBADmycHisA vector, indicate protein binding to the promoter of interest.

### Pull-down assays

For bacterial co-expression experiments, genes encoding NsrR, Crp or Crl were cloned into the first multiple cloning site of pCDFDuet-1 vector (Novagen) allowing expression of the proteins tagged with a N-terminal hexahistidine motif. Genes encoding RpoA or RpoS were cloned into the second multiple cloning site using PCR primers allowing the insertion of a N-terminal HA motif (see Table S2 for primers). *E. coli* BL21(DE3) harboring the different constructs was grown at 37°C to OD_600 nm_ of 0.7, then induced with 1 mM IPTG and grown for an additional 2 h. After resuspension of bacteria with a 1/10e volume of lysis buffer (50 mM NaH_2_PO_4_, 300 mM NaCl), samples were sonicated and centrifuged. Supernatants (whole bacterial extracts) were incubated with Ni-NTA beads at 4°C for 16 h. Beads were washed four times with lysis buffer containing 60 mM imidazole and bound proteins were eluted with lysis buffer containing 250 mM imidazole.

### Bacterial mRNA analysis

Total RNA from bacteria was extracted using the TRI Reagent RNA Isolation Reagent (Sigma). Each RNA sample (1 µg) was reverse transcribed with Superscript II enzyme (Invitrogen) and random primers (Invitrogen). The cDNAs and serial dilutions of EDL933 genomic DNA, which were used for the standard curves, were amplified with gene-specific primers (Table S2) in the Eppendorf Mastercycler ep*realplex* (Eppendorf) apparatus. The results are presented as the ratios between the copy number of mRNA of the gene of interest and the copy number of *rpoA* mRNA.

### Western-blot analysis

Samples were mixed with a 2X SDS-PAGE sample buffer, heated for 5 min at 100°C, resolved on 14% SDS-PAGE gels and blotted on PVDF membranes. Membranes were blocked in PBS-0.05% Tween 20 supplemented with 5% non-fat dry milk, then probed with murine monoclonal anti-HA or HRP-conjugated anti-HIS Abs (Sigma; 1/4000 for each). An HRP-conjugated goat anti-murine IgG Ab (Sigma) was also used for the HA blots. Acquisitions were performed with a G:box system (Syngene).

### Cell cultures and infections

The epithelial cell lines Hct-8 and HeLa were maintained in DMEM with 10% FCS, 10 mM Hepes, 100 U/ml penicillin, 100 µg/ml streptomycin at 37°C under 5% CO_2_. Hct-8 cells were plated on LabTek (Nunc), cultured for 7 days, and stimulated for 24 h with human IFN-γ (50 ng/ml), TNF-α (20 ng/ml), and IL-1β (5 ng/ml). HeLa cells were seeded into LabTek and grown for 24 h. These Hct-8 and HeLa cells were washed, and infected with bacteria with an MOI of 100, in the presence or absence of NOR-4 or of the iNOS inhibitor l-NIL. After 4 washes with PBS, cells were fixed using 1 ml methanol for 15 min at −20°C and stained with Giemsa or May-Grünwald Giemsa for 30 min. The number of adherent bacteria per cell was counted using the AxioVision 4 software.

### Determination of NO concentration

The concentration of the stable oxidized products of NO, NO_3_^−^ and NO_2_^−^, was measured using the Nitrite/Nitrate Assay Kit (Cayman Chemical).

### Statistics

All the data represent the mean ± SEM. Student's t test or ANOVA with the Newman-Keuls test were used to determine significant differences between two groups or to analyze significant differences among multiple test groups, respectively.

## Supporting Information

Figure S1 **Kinetic of NO released by NOR-4.** The concentrations of NO_3_^−^+NO_2_^−^ were determined in DMEM medium containing 500 µM NOR-4.
(TIF)

Figure S2 **The promoter regions of** ***gadE*** **,** ***gadX*** **, LEE1, LEE4, and LEE5 in the EHEC strain EDL933.** Arrows indicate the location of the primers used for the ChIP experiments.
(TIF)

Figure S3 **Conservation of the putative NsrR binding site in the LEE1, LEE4 and LEE5 promoters of selected EHEC, EPEC, and** ***C. rodentium*** **strains.** *P* indicates the probability that the sequence be an NsrR binding site, as determined by the online software Gibbs Motif Sampler; ns, not significant.
(TIF)

Table S1 **Bacterial strains and plasmids.**
(DOCX)

Table S2 **List of primers.**
(DOCX)
