# Supplementary figures and images for: NsrR, GadE, and GadX Interplay in Repressing Expression of the Escherichia coli O157:H7 LEE Pathogenicity Island in Response to Nitric Oxide
Source: PLoS Pathog. 2014 Jan 9;10(1):e1003874. doi: 10.1371/journal.ppat.1003874 (PMC3887101; doi:10.1371/journal.ppat.1003874)

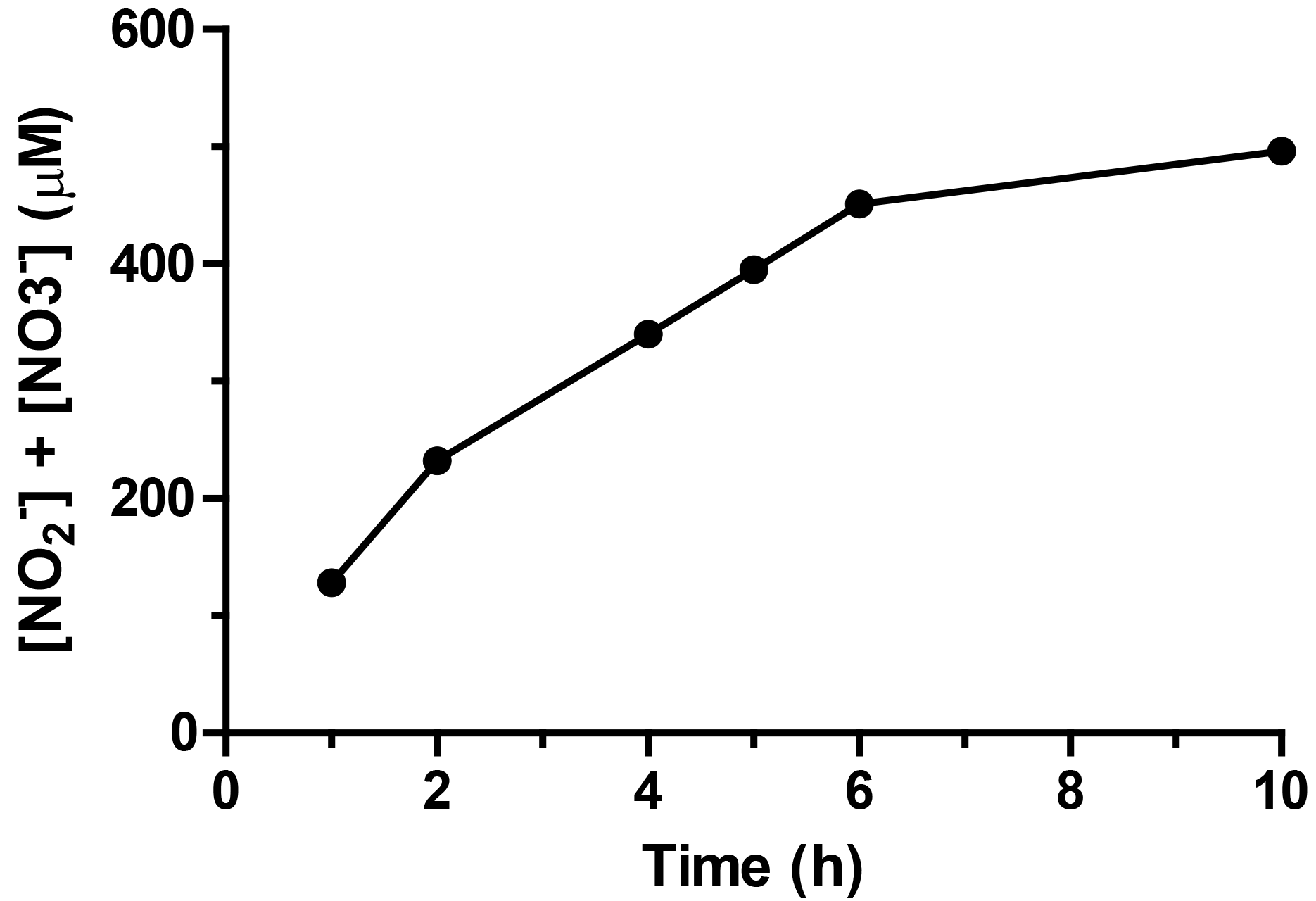

Supplement: Figure S1 — Kinetic of NO released by NOR-4. The concentrations of NO3 −+NO2 − were determined in DMEM medium containing 500 µM NOR-4. (TIF) [file ppat.1003874.s001.tif]

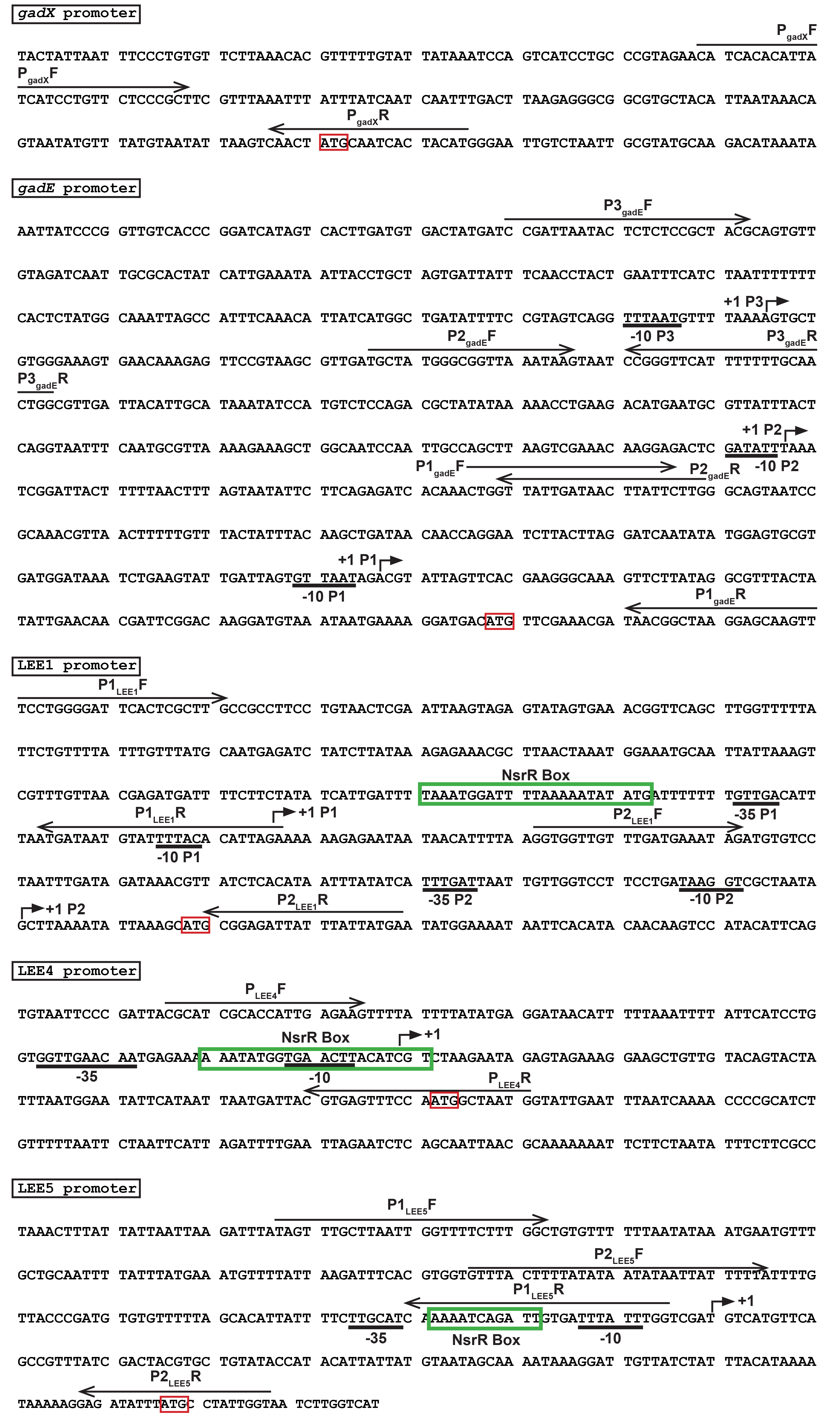

Supplement: Figure S2 — The promoter regions of gadE , gadX , LEE1, LEE4, and LEE5 in the EHEC strain EDL933. Arrows indicate the location of the primers used for the ChIP experiments. (TIF) [file ppat.1003874.s002.tif]

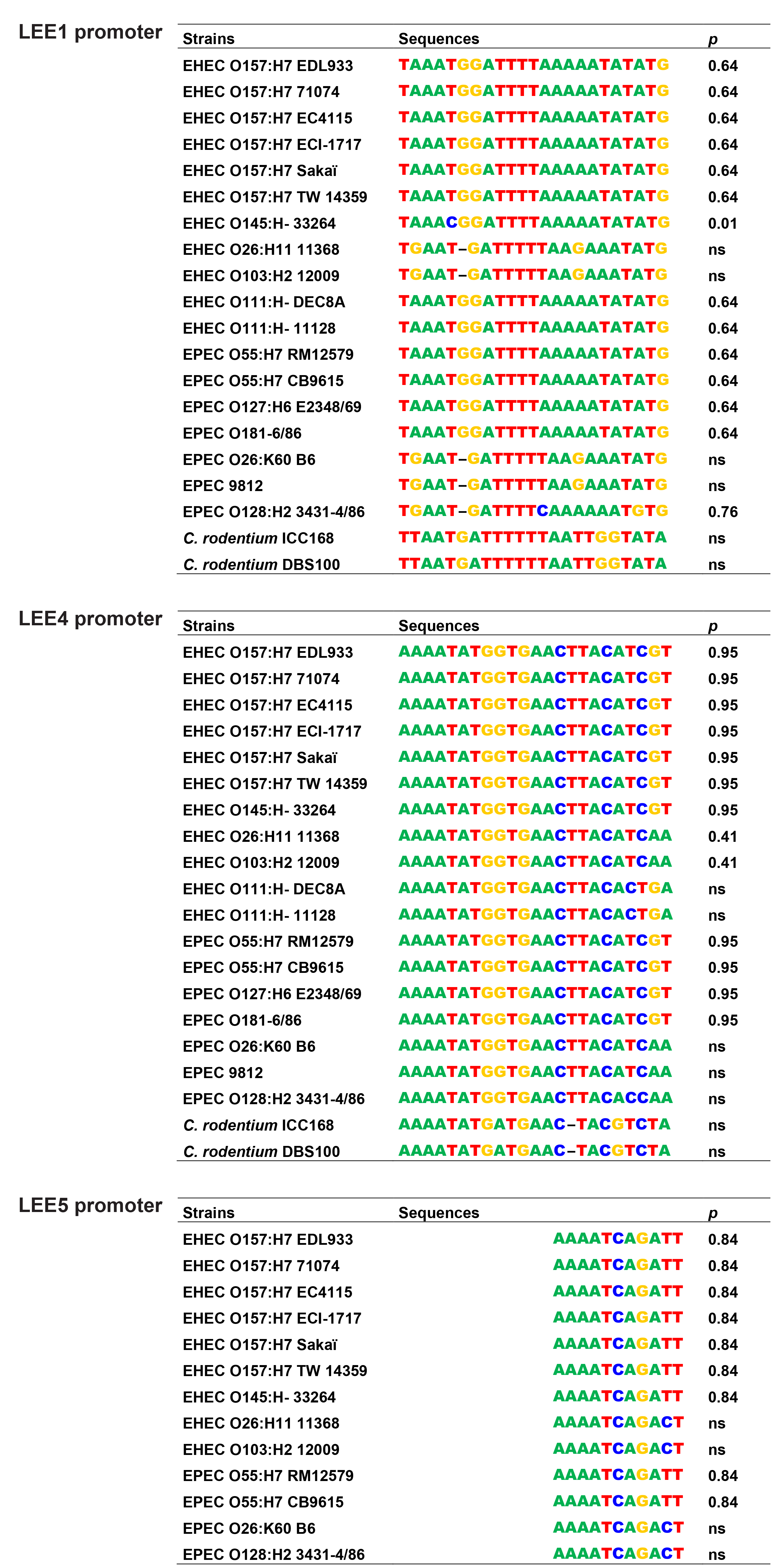

Supplement: Figure S3 — Conservation of the putative NsrR binding site in the LEE1, LEE4 and LEE5 promoters of selected EHEC, EPEC, and C. rodentium strains. P indicates the probability that the sequence be an NsrR binding site, as determined by the online software Gibbs Motif Sampler; ns, not significant. (TIF) [file ppat.1003874.s003.tif]
