# Supplementary material for: NsrR, GadE, and GadX Interplay in Repressing Expression of the Escherichia coli O157:H7 LEE Pathogenicity Island in Response to Nitric Oxide
Source: PLoS Pathog. 2014 Jan 9;10(1):e1003874. doi: 10.1371/journal.ppat.1003874 (PMC3887101; doi:10.1371/journal.ppat.1003874)
Supplement: Table S1 — Bacterial strains and plasmids. (DOCX) [file ppat.1003874.s004.docx]

**Supplementary Table S1: Bacterial strains and plasmids**

| **Strains and plasmids** | **Relevant characteristics** | **References** |
| --- | --- | --- |
| **Strains** |  |  |
| EDL933 | wild type EHEC O157:H7 | [^1^](#_ENREF_1) |
| Δ*gadE* | EDL933 Δ*gadE* CamR | This work |
| Δ*gadX* | EDL933 Δ*gadX* CamR | This work |
| Δ*gadE*/*gadX* | EDL933 Δ*gadE*∆*gadX* CamR KanR | This work |
| Δ*nsrR* | EDL933 Δ*nsrR* KanR | [^2^](#_ENREF_2) |
| BL21 (DE3) | laboratory *E. coli* | Stratagene |
| **Plasmids** |  |  |
| pBAD33 | low copy P*ara* CamR | [^3^](#_ENREF_3) |
| pBADMycHisA | low copy P*ara* 6His tagged C-ter AmpR | Invitrogen |
| pBADHisA | low copy P*ara* 6His tagged N-ter AmpR | Invitrogen |
| p*gadE* | pBADMycHisA::*gadE* | This work |
| p*gadX* | pBADHisA::*gadX* | This work |
| p*nsrR* | pBADMycHisA::*nsrR* | This work |
| p33*nsrR* | pBAD33::*nsrR* | This work |
| pCDFDuet-1 | vector designed for coexpresssion of two target proteins | Novagen |
| pX_HA-RpoA | pCDFDuet-1 expressing HA-RpoA | This work |
| pHis-NsrR_HA-RpoA | pCDFDuet-1 expressing HA-RpoA and His-NsrR | This work |
| pHis-Crp_HA-RpoA | pCDFDuet-1 expressing HA-RpoA and His-Crp | This work |
| pX_HA-RpoS | pCDFDuet-1 expressing HA-RpoS | This work |
| pHis-NsrR_HA-RpoS | pCDFDuet-1 expressing HA-RpoS and His-NsrR | This work |
| pHis-Crl_HA-RpoS | pCDFDuet-1 expressing HA-RpoS and His-Crl | This work |

O'Brien, A.O., Lively, T.A., Chen, M.E., Rothman, S.W., and Formal, S.B. (1983) *Escherichia coli* 0157:H7 strains associated with haemorrhagic colitis in the United States produce a *Shigella dysenteriae* 1 (SHIGA) like cytotoxin. *Lancet* **1:** 702.

Vareille, M., de Sablet, T., Hindre, T., Martin, C., and Gobert, A.P. (2007) Nitric oxide inhibits Shiga-toxin synthesis by enterohemorrhagic *Escherichia coli*. *Proc Natl Acad Sci USA* **104:** 10199-10204.

Guzman, L.M., Belin, D., Carson, M.J., and Beckwith, J. (1995) Tight regulation, modulation, and high-level expression by vectors containing the arabinose PBAD promoter. *J Bacteriol* **177:** 4121-4130.
