# Supplementary material for: NsrR, GadE, and GadX Interplay in Repressing Expression of the Escherichia coli O157:H7 LEE Pathogenicity Island in Response to Nitric Oxide
Source: PLoS Pathog. 2014 Jan 9;10(1):e1003874. doi: 10.1371/journal.ppat.1003874 (PMC3887101; doi:10.1371/journal.ppat.1003874)
Supplement: Table S2 — List of primers. (DOCX) [file ppat.1003874.s005.docx]

**Supplementary Table S2: List of primers**

| **Sequences** | **Use** | **References** |
| --- | --- | --- |
| F: ATGATTTTTCTCATGACGAAAGATTCTTTTCT TTTACAGGGCTTTTGGCAgtgtaggctggagctgcttc | Construction of *gadE* mutant | This work |
| R: CTAAAAATAAGATGTGATACCCAGGGTGACGA TGTCGCTCATACGTCTTAcatatgaatatcctccttagt |  |  |
| F: GCGTGCTACATTAATAAACAGTAATATGTTTA TGTAATATTAAGTCAACTgtgtaggctggagctgcttc | Construction of *gadX* mutant | This work |
| R: TCCTCTTCCCGGTCCCCTATGCCGGGTTTTTTTT ATGTCTGAGTAAAACTcatatgaatatcctccttagt |  |  |
| F: GCGTGCTACATTAATAAACAGTAATATGTTTATG TAATATTAAGTCAACTagccacgttgtgtctcaaaatc | Construction of *gadX* mutant in the *gadE* mutant | This work |
| R: TCCTCTTCCCGGTCCCCTATGCCGGGTTTTTTTTA TGTCTGAGTAAAACTttagaaaaactcatcgagca |  |  |
| F: CATGCCATGGCCATGTTCGAAACGATAACG | *gadE* cloning in pBADMycHisA | This work |
| R: CCCAAGCTTAAAATAAGATGTGATACCCAG |  |  |
| F: CCGCTCGAGCAATCATTACATGGGAATTG | *gadX* cloning in pBADHisA | This work |
| R: CCCAAGCTTGGAGACGGCAGACTATCCT |  |  |
| F: CCGAGCTCGAGACAGTTAACGAGTTTCACTGAT | *nsrR* cloning in pBADMycHisA | This work |
| R: CCCAAGCTTCTCCACCAGCAATAATTTATA |  |  |
| F: GAAGATCTCTACCCATACGACGTCCCAGACTA CGCTCAGGGTTCTGTGACAGAGTTTC | *rpoA* cloning in pCDFDuet-1 | This work |
| R:CCGCTCGAGTTACTCGTCAGCGATGCTTG |  |  |
| F:GAAGATCTCTACCCATACGACGTCCCAGACTACGCTAGTCAGAATACGCTGAAAGTTCATG | *rpoS* cloning in pCDFDuet-1 | This work |
| R: CCGCTCGAGTTACTCGCGGAACAGCGCTTC |  |  |
| F:CGGGATCCGCAGTTAACGAGTTTCACTGATTACGG | *nsrR* cloning in pX_HA-RpoA or pX_HA-RpoS | This work |
| R: ATAAGAATGCGGCCGCTCACTCCACCAGCAATA ATTTATAAAGC |  |  |
| F: CGGGATCCGGTGCTTGGCAAACCGCAAAC | *crp* cloning in pX_HA-RpoA | This work |
| R: ATAAGAATGCGGCCGCTCACTCCACCAGCAATA ATTTATAAAGC |  |  |
| F: CGGGATCCGACGTTACCGAGTGGACACCCG | *crl* cloning in pX_HA-RpoA | This work |
| R: ATAAGAATGCGGCCGCTCACGCCGTTAACTTCAC CGG |  |  |
| F: GACTGCGAGAGCAGGAAGTT | *ler* mRNA quantification | This work |
| R: GAGTCCATCATCAGGCACATT |  |  |
| F: GGAACAAATCGCACCGTTAG | *sepZ* mRNA quantification | This work |
| R: ATTCTGTGCTGCTCGTCTCC |  |  |
| F: GGGAGCTGATTTGTCTAATAG | *escV* mRNA quantification | This work |
| R: GCATGACCTCAATTTGCTGTG |  |  |
| F: CGAAAGAAGCGTTCCAGAAC | *tir* mRNA quantification | This work |
| R: CTGCTGCTTTAGCCTGCTCT |  |  |
| F: CGTTCTGTCGAATGGTCAAG | *eae* mRNA quantification | This work |
| R: CACCGTCGCGGTATAAGTAA |  |  |
| F: CGGCACAAAAGATGGCTAAT | *espA* mRNA quantification | This work |
| R: ACCAGCGCTTAAATCACCAC |  |  |
| F: GAGAAATTAGATGCCGAGAG | *gadE* mRNA quantification | This work |
| R: TTGTGAATTCTTATGGGGCA |  |  |
| F: CGAGCTGTTGATGAGTCCAA | *gadX* mRNA quantification | This work |
| R: GCAAAGCACGTTGCATTCTA |  |  |
| F: GGTGAGAGTTCAGGGCAAAG | *rpoA* mRNA quantification | This work |
| R: ACCGCGCTGAACTTTGATAC |  |  |
| P1_LEE1_F: TCCTGGGGATTCACTCGCTTG | P1 LEE1 promoter quantification | Kendall *et al.*, |
| P1_LEE1_R: TCTAATGTGTAAAATACAT |  | 2010 |
| P2_LEE1_F: GGTGGTTGTTTGATGAAATAG | P2 LEE1 promoter quantification | This work |
| P2_LEE1_R: TCATAATAAATAATCTCCGC |  |  |
| P_LEE4_F: CGCATCGCACCATTGAGAAG | LEE4 promoter quantification | This work |
| P _LEE4_R: CATTAGCCATTGGAAACTCACG |  |  |
| P1_LEE5_F: ATAGTTTGCTTAATTGGTTTTCTTTG | P1 LEE5 promoter quantification | This work |
| P1_LEE5_R: CCAAATAAATCACAATCTGATTTTTG |  |  |
| P2_LEE5_F: GTTTACTTTTATATAATATAATTATTTTT | P2 LEE5 promoter quantification | This work |
| P2_LEE5_R: ACCAATAGGCATAAATATCTCC |  |  |
| P_gadX_F: CATCACACATTATCATCCTGTTCTCCCGCT | *gadX* promoter quantification | Hommais *et al.*, |
| P_gadX_R: ATGTAGTGATTGCATAGTTG |  | 2004 |
| P1_gadE_F: CTGGTTATTGATAACTTATTC | P1 *gadE* promoter quantification | Tree *et al.*, 2011 |
| P1_gadE_R: AACTTGCTCCTTAGCCGTTA |  |  |
| P2_gadE_F: TGCTATGGGCGGTTAAATAAG | P2 *gadE* promoter quantification | Tree *et al.*, 2011 |
| P2_gadE_R: CAAGAATAAGTTATCAATAAC |  |  |
| P3_gadE_F: CCGATTAATACTCTCTCCGCTACG | P3 *gadE* promoter quantification | This work |
| P3_gadE_R: CCAGTTGCAAAAAAATGAACCCGG |  |  |
| P_gadA_F GAACTCCTTAAATTTATTTG | *gadA* promoter quantification | Hommais *et al*., |
| P_gadA_R: TTTGGGCGATTTTTATTACG |  | 2004 |
| P_hmpA_F: GAACCATTTACATTGCAGGGC | *hmpA* promoter quantification |  |
| P_hmpA_R: CGTCAAGCATATGGTCTTCC |  |  |

Hommais, F., Krin, E., Coppee, J.Y., Lacroix, C., Yeramian, E., Danchin, A.*, et al.* (2004) GadE (YhiE): a novel activator involved in the response to acid environment in *Escherichia coli*. *Microbiology* **150**: 61-72.

Kendall, M.M., Rasko, D.A., and Sperandio, V. (2010) The LysR-type regulator QseA regulates both characterized and putative virulence genes in enterohaemorrhagic *Escherichia coli* O157:H7. *Mol Microbiol* **76**: 1306-1321.

Tree, J.J., Roe, A.J., Flockhart, A., McAteer, S.P., Xu, X., Shaw, D.*, et al.* (2011) Transcriptional regulators of the GAD acid stress island are carried by effector protein-encoding prophages and indirectly control type III secretion in enterohemorrhagic *Escherichia coli* O157:H7. *Mol Microbiol* **80**: 1349-1365.
